# Distinct Features of Canine Non-conventional CD4^−^CD8α^−^ Double-Negative TCRαβ^+^ vs. TCRγδ^+^ T Cells

**DOI:** 10.3389/fimmu.2019.02748

**Published:** 2019-11-22

**Authors:** Friederike V. Rabiger, Kathrin Rothe, Heiner von Buttlar, Doris Bismarck, Mathias Büttner, Peter F. Moore, Maria Eschke, Gottfried Alber

**Affiliations:** ^1^Institute of Immunology/Molecular Pathogenesis, Center for Biotechnology and Biomedicine, College of Veterinary Medicine, University of Leipzig, Leipzig, Germany; ^2^Department of Pathology, Microbiology and Immunology, School of Veterinary Medicine, University of California, Davis, Davis, CA, United States

**Keywords:** dog, canine T cells, CD4^−^CD8α^−^, double-negative, non-conventional T cells, TCRαβ, TCRγδ

## Abstract

The role of conventional TCRαβ^+^CD4^+^ or TCRαβ^+^CD8α^+^ single-positive (sp) T lymphocytes in adaptive immunity is well-recognized. However, non-conventional T cells expressing TCRαβ or TCRγδ but lacking CD4 and CD8α expression [i.e., CD4^−^CD8α^−^ double-negative (dn) T cells] are thought to play a role at the interface between the innate and adaptive immune system. Dn T cells are frequent in swine, cattle or sheep and predominantly express TCRγδ. In contrast, TCRγδ^+^ T cells are rare in dogs. In this study, we identified a high proportion of canine dn T cells in the TCRαβ^+^ T cell population of PBMC, lymphatic and non-lymphatic organs. In PBMC, the frequency of this T cell subpopulation made up one third of the frequency of TCRαβ^+^CD4^+^ sp, and almost half of the frequency of TCRαβ^+^CD8α^+^ sp T cells (i.e., ~15% of all TCRαβ^+^ T cells). Among TCRαβ^+^CD4^−^CD8α^−^ dn T cells of PBMC and tissues, FoxP3^+^ cells were identified indicating regulatory potential of this T cell subset. 80% of peripheral blood FoxP3^+^TCRαβ^+^CD4^−^CD8α^−^ dn T cells co-expressed CD25, and, interestingly, also the FoxP3-negative TCRαβ^+^CD4^−^CD8α^−^ dn T cells comprised ~34% CD25^+^ cells. Some of the FoxP3-positive TCRαβ^+^CD4^−^CD8α^−^ dn T cells co-expressed GATA-3 suggesting stable function of regulatory T cells. The frequency of GATA-3 expression by FoxP3^−^TCRαβ^+^CD4^−^CD8α^−^ dn T cells was even higher as compared with TCRαβ^+^CD4^+^ sp T cells (20.6% vs. 11.9%). Albeit lacking FoxP3 and CD25 expression, TCRγδ^+^CD4^−^CD8α^−^ dn T cells also expressed substantial proportions of GATA-3. In addition, TCRαβ^+^CD4^−^CD8α^−^ dn T cells produced IFN-γ and IL-17A upon stimulation. T-bet and granzyme B were only weakly expressed by both dn T cell subsets. In conclusion, this study identifies two dn T cell subsets in the dog: (i) a large (~7.5% in Peyer's patches, ~15% in lung) population of TCRαβ^+^CD4^−^CD8α^−^ dn T cells with subpopulations thereof showing an activated phenotype, high expression of FoxP3 or GATA-3 as well as production of IFN-γ or IL-17A and (ii) a small TCRγδ^+^CD4^−^CD8α^−^ dn T cell subset also expressing GATA-3 without production of IFN-γ or IL-17A. It will be exciting to unravel the function of each subset during immune homeostasis and diseases of dogs.

## Introduction

Dogs are important companion animals which develop a range of immune-mediated diseases such as allergies, cancer (e.g., mammary tumors), or autoimmune disorders that are very similar to those occurring in the human species (1, 2). With the dog living in close contact with people, it is not only worth studying these diseases for the dog itself, but it might also be a useful model to draw conclusions for humans. For this reason, it is essential to intensify research on the canine immune system which is still poorly understood.

Besides the well-known conventional single-positive (sp) T cells (i.e., CD4^+^ and CD8α^+^ sp T cells) there are extrathymic non-conventional CD4^−^CD8α^−^ double-negative (dn) T cells lacking the CD4 and CD8α co-receptors (3). These cells were described almost 30 years ago for man and mice (4, 5). Interestingly, even earlier “unusual subpopulations” of T cells missing expression of CD4 and CD8α were observed in swine (6, 7) and subsequently identified as TCRγδ^+^ T cells (8, 9). In sheep, cattle, and chicken also early immunological studies unraveled the existence of high numbers of blood and tissue TCRγδ^+^ T cells which were mostly CD4^−^CD8α^−^ dn T cells (10–14). With this pioneering research in veterinary immunology the concept of “γδ T cell high” species (e.g., swine, sheep, cattle, chicken) and “γδ T cell low” species (man, mouse) was established (15). Thus, comparative immunology has contributed to a broader and deeper view into the nature of non-conventional T cell populations, especially for γδ T cells. Studies looking at canine non-conventional lymphocyte subsets started later and characterized the dog as a “γδ T cell low” species (16). More recently, questions about the occurrence, regulation and function of CD4^−^CD8α^−^ dn T cells were raised in context with the investigation of regulatory T cell populations of healthy dogs (17), canine leishmaniasis (18), or upon specific immunotherapy for dogs with adverse food reactions (19).

For more extended functional aspects of CD4^−^CD8α^−^ dn T cells, of course, murine and human systems with ample reagents available proofed to be more accessible than research in domestic animals, albeit at the cost of a narrower scientific perspective. Thus, studies on CD4^−^CD8α^−^ dn T cell functions done in rodents and humans demonstrated immunoregulatory activity and a role in autoimmunity as reviewed recently (3, 20). Based on their regulatory potential, murine and human CD4^−^CD8α^−^ dn T cells have been termed “non-conventional regulators” (21).

Research on CD4^−^CD8α^−^ dn T cells of domestic animals nevertheless proceeded either driven by the generation of new monoclonal antibodies against species-specific markers relevant for research on CD4^−^CD8α^−^ dn T cells or by the identification of cross-reactive antibodies. Functional features of γδ T cells have been characterized in cattle where expression of WC1, a member of the CD163 family, was shown to act as a γδ T cell co-stimulatory receptor and pattern recognition receptor (PRR) for pathogenic bacteria (22, 23). In swine, a recent functional analysis of γδ T cells revealed distinct expression patterns of transcription factors and cytokines depending on the γδ T cell phenotypes (24). In dog, extrathymic non-conventional CD4^−^CD8α^−^ dn T cells (CD3^+^, TCRαβ^+^, or TCRγδ^+^) have only been described in single studies (18, 19, 25) and a comprehensive characterization of these cells is still missing. Thus, we chose to perform a systematic multiparameter flow cytometry analysis of canine CD4^−^CD8α^−^ dn T cells. We found surprisingly high proportions of CD4^−^CD8α^−^ dn T cells in peripheral blood mononuclear cells (PBMC), lymphoid and non-lymphoid organs of healthy dogs. It is noteworthy that the majority of canine CD4^−^CD8α^−^ dn T cells is TCRαβ^+^, with ~ 1/3 expressing the activation marker CD25 and a substantial part of those CD25^+^ cells expressing FoxP3. Subpopulations of these TCRαβ^+^CD4^−^CD8α^−^ dn cells also express GATA-3 and produce almost comparable amounts of IFN-γ or IL-17A as their CD4^+^ sp counterparts. On the other hand, they express only low frequencies of T-bet and granzyme B. In contrast, the small TCRγδ^+^ CD4^−^CD8α^−^ dn T cell population does neither express markers of activation nor FoxP3, IFN-γ or IL-17A, but resembles its TCRαβ^+^ counterpart regarding the high frequencies of GATA-3, and low frequencies of T-bet and granzyme B expressing cells.

By comparing immune cell subpopulations across different species, we gain a broader and deeper view into the nature and function of these cells, which ultimately will lead to the identification of the most suitable animal species serving as model for human diseases.

## Materials and Methods

### Animals, Blood, and Tissue Samples

Venous blood was taken from 10 healthy experimental Beagle dogs (five female, five male, age: 3–9 years) into heparinized vacutainer tubes (BD Vacutainer®, 10 ml, Li-Heparin 17 IU/ml, Becton Dickinson, Heidelberg, Germany). At the time of blood sampling, the dogs belonged to the College of Veterinary Medicine, University of Leipzig, Germany. The Animal Care and Usage Committee of the Saxony State Office (*Landesdirektion Sachsen*) in Leipzig, Germany, authorized the study (approval numbers: A 10/14 and A 28/18).

Tissue samples were collected from another group of experimental Beagle dogs (Marshall Bioresources, North Rose, NY, USA, *n* = 12, six female, six male, age: 10–15 months). The dogs were clinically healthy animals which were euthanized for reasons unrelated to our studies (control group of an animal experiment for preclinical drug development, approval number V54-19c 20/15-DA4/Anz.1004). Necropsies and histopathological examinations confirmed the physical health of every single dog. Following euthanasia, full thickness sections from mesenteric (mLN) and tracheobronchial (tLN) lymph nodes, spleen, duodenum, jejunum, and lung were collected immediately for further processing (mLN, spleen, duodenal/jejunal Peyer's patches, lung: *n* = 10, tLN: *n* = 9).

### Isolation of Peripheral Blood Mononuclear Cells (PBMC)

Whole blood was diluted in phosphate buffered saline (PBS) at a ratio of 1:1, layered above Biocoll Separating Solution (Biochrom AG, Berlin, Germany) and centrifuged at 500 × g for 30 min at room temperature (RT). After washing with PBS, cells were treated with erythrocyte lysis buffer (150 mM NH_4_Cl, 8 mM KHCO_3_, 2 mM EDTA; pH 7) for 5 min at RT and the lysis reaction was stopped with PBS containing 3% fetal bovine serum (FBS, Thermo Fisher Scientific, Carlsbad, USA; and PAN-Biotech, Aidenbach, Germany). Next, PBMC were washed with PBS and counted with a microscope using a hemocytometer (Laboroptik, Lancing, UK) and trypan blue (Sigma-Aldrich, Taufkirchen, Germany).

### Stimulation of PBMC

PBMC were resuspended in RPMI 1640 medium (Biochrom, Berlin, Germany) containing 100 U/ml penicillin, 100 μg/ml streptomycin (both purchased from PAA Laboratories), and 10% FBS (Thermo Fisher Scientific, Carlsbad, USA). Cells were cultured overnight (37°C, 5% CO_2_) at a density of 5 × 10^5^ cells per well in 96 well flat bottom plates (TPP Techno Plastic Products AG, Trasadingen, Switzerland). Stimulation was done the next day with 0.22 μg/ml phorbol-myristate-acetate (PMA)/ionomycin for 4 h in combination with 5 μg/ml Brefeldin A. Medium incubation served as negative control.

### Generation of Single Cell Suspensions of Lymph Nodes and Spleen

Leukocytes from mLN, tLN, and spleen were isolated as previously described (26). In brief, tissue pieces were minced, passed through a 100 μm nylon cell strainer (BD Biosciences, Heidelberg, Germany) and resuspended in PBS followed by lysis of erythrocytes and cell counting as mentioned earlier.

### Isolation of Lymphocytes From Peyer's Patches

After collection from duodenum and jejunum, Peyer's Patches (PP) were immediately washed in ice-cold Hank's Balanced Salt Solution (HBSS) without Ca^2+^ and Mg^2+^ (PAN-Biotech, Aidenbach, Germany) supplemented with 10 mM HEPES (Carl Roth, Karlsruhe, Germany) and 2% FBS (Thermo Fisher Scientific, Carlsbad, USA; and PAN-Biotech, Aidenbach, Germany). Afterwards, the pieces were incubated 3 × 30 min in HBSS containing 2 mM 1,4-Dithioerythritol (DTE, Sigma-Aldrich, Taufkirchen, Germany), and 0.5 mM EDTA (Carl Roth, Karlsruhe, Germany) at 37°C under continuous stirring to remove the intestinal epithelium. The remaining tissue was minced, dissociated by the gentleMACS™ Dissociator (Miltenyi Biotec, Bergisch Gladbach, Germany), and passed through a 100 μm cell strainer (BD Biosciences, Heidelberg, Germany). To purify lymphocytes, cells were centrifuged on a discontinuous density gradient with 40% and 70% Percoll (900 × g, 20 min, RT), harvested at the interphase, and washed in RPMI 1640 medium (Biochrom, Berlin, Germany) containing 5% FBS and 50 μg/ml Gentamicin (Sigma-Aldrich, Taufkirchen, Germany; and PAN-Biotech, Aidenbach, Germany).

### Isolation of Lung Leukocytes

Lung tissue was cut into small pieces and digested for 30 min at 37°C in RPMI 1640 medium (Biochrom, Berlin, Germany) supplemented with DNase I (111 U/ml; Sigma-Aldrich, Taufkirchen, Germany), Collagenase D (0.7 mg/ml; Roche Diagnostics Deutschland GmbH, Mannheim, Germany), and 1 mM sodium pyruvate (AppliChem, Darmstadt, Germany). Following passage through 100 μm cell strainers (BD Biosciences, Heidelberg, Germany), erythrocytes were lysed as described above. Leukocytes were separated from tissue cells by 30%/70% Percoll (GE Healthcare, Uppsala, Sweden) gradient centrifugation (400 × g, 20 min, RT). Cells were recovered from the interphase and resuspended in IMDM medium (PAN-Biotech, Aidenbach, Germany) supplemented with 100 U/ml penicillin, 100 μg/ml streptomycin (both purchased from PAA Laboratories), and 10% FBS (Thermo Fisher Scientific, Carlsbad, USA; and PAN-Biotech, Aidenbach, Germany).

### Flow Cytometric Analysis

Fixable viability dye eFluor 780 (Thermo Fisher Scientific, Carlsbad, USA) was used according to the manufacturer's protocol to discriminate dead from viable cells. In a second step, they were incubated with a mixture of heat-inactivated normal serum derived from dog, rat, and mouse (each 15% in PBS) to block non-idiotypic binding. Next, surface staining was performed by incubating cells with primary antibodies for 15 min in the dark on ice. To detect canine TCRαβ and TCRγδ, a PerCP/Cy5.5-conjugated goat-anti-mouse IgG secondary antibody (Biolegend, San Diego, USA) was used. In this case, blockade of non-idiotypic binding was performed with a mixture of heat-inactivated rat, dog and goat normal serum (each 15% in PBS). If CD25 was included in the surface staining panel, incubation with the P4A10 antibody derived from mice was performed separately after an additional blocking step including mouse serum to saturate possible free binding sites of the goat-anti-mouse secondary antibody. The details of all primary antibodies used for flow cytometric staining are summarized in Table 1. Cross-reactivity of antibodies directed against non-canine antigens was validated by several groups (see references in Table 1) or reported by the supplier (e.g., anti-CD3ε clone CD3-12). For the anti-human/mouse GATA-3 antibody TWAJ, cross-reactivity with canine GATA-3 is very likely based on *in silico* epitope prediction by Kolaskar and Tongaonkar 1990 which leads to nearly identical epitopes predicted in the murine, canine and human sequence (37). Moreover, the homology of canine to murine GATA-3 is very high (above 95%).

**Table 1:** Primary antibodies used for flow cytometry.

**Antigen**	**Clone**	**Species reactivity**	**Isotype**	**Source**	**Fluorochrome**	**References**
TCRαβ	CA15.8G7	canine	Mouse IgG1	Leukocyte Antigen Biology Laboratory, Davis, USA	Hybridoma supernatant	not applicable (N/A)
TCRγδ	CA20.8H1	canine	Mouse IgG2a	Leukocyte Antigen Biology Laboratory, Davis, USA	Hybridoma supernatant	N/A
CD4	YKIX302.9	canine	Rat IgG2a	Thermo Fisher Scientific, Carlsbad, USA Bio-Rad, Munich, Germany	APC Pacific Blue, RPE	N/A
CD8α	YCATE55.9	canine	Rat IgG1	Thermo Fisher Scientific, Carlsbad, USA Bio-Rad, Munich, Germany	APC, PerCP-eFluor 710 Pacific Blue, Alexa Fluor 647	N/A
CD25	P4A10	canine	Mouse IgG1	Thermo Fisher Scientific, Carlsbad, USA	PE	N/A
FoxP3	FJK-16s	mouse/rat	Rat IgG2a	Thermo Fisher Scientific, Carlsbad, USA	FITC	(26–29)
GATA-3	TWAJ	human/mouse	Rat IgG2b	Thermo Fisher Scientific, Carlsbad, USA	eFluor 660	–
T-bet	eBio4B10	human/mouse	Mouse IgG1	Thermo Fisher Scientific, Carlsbad, USA	eFluor 660	(27, 30)
IFN-γ	CC302	bovine	Mouse IgG1	Bio-Rad, Munich, Germany	RPE	(27, 31–33)
IL-17A	eBio64DEC17	human	Mouse IgG1	Thermo Fisher Scientific, Carlsbad, USA	Alexa Fluor 488	(34, 35)
Granzyme B	GB11	human/mouse	Mouse IgG1	Biolegend, San Diago, USA	FITC	(26, 27)
CD5	YKIX322.3	canine	Rat IgG2a	Thermo Fisher Scientific, Carlsbad, USA	PE, PerCP-eFluor 710	N/A
CD3	CD3-12	human	Rat IgG1	Bio-Rad, Munich, Germany	FITC	(36)

**Figure 1:**
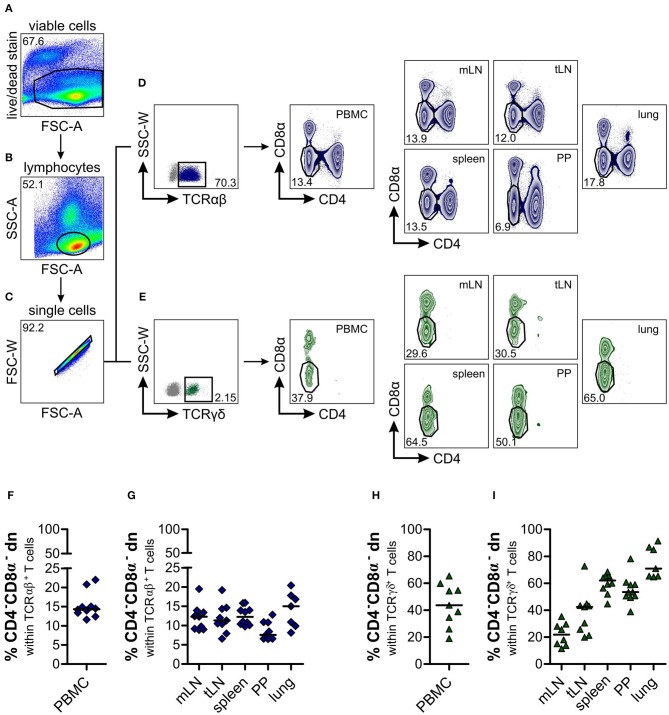
Identification of a substantial proportion of TCRαβ^+^CD4^−^CD8α^−^ double-negative (dn) T cells in different organs of dogs. Leukocytes from lymphatic and non-lymphatic organs of healthy Beagle dogs (*n* = 7–10), i.e., peripheral blood mononuclear cells (PBMC), mesenteric lymph nodes (mLN), tracheobronchial lymph nodes (tLN), spleen, Peyer‘s patches (PP), and lung were analyzed by multicolor flow cytometry. Viable lymphocytes **(A,B)** were gated after doublet-exclusion **(C)** on TCRαβ^+^ (**D**, left panel) resp. TCRγδ^+^ (**E**, left panel) T cells. Representative zebra plots of TCRαβ^+^CD4^−^CD8α^−^ dn T cells are shown in dark blue (**D**, middle and right panels), green zebra plots show TCRγδ^+^CD4^−^CD8α^−^ dn T cells of organs indicated above (**E**, middle and right panels). Numbers in plots imply percentages. **(F–I)** Quantification of TCRαβ^+^CD4^−^CD8α^−^ (**F, G**; dark blue diamonds) and TCRγδ^+^CD4^−^CD8α^−^ dn T cells (**H, I**; green triangles) in indicated organs. Each symbol represents one individual dog, the horizontal bars display median values. Peripheral blood (*n* = 9–10) was taken from a different group of Beagle dogs than tissues (*n* = 7–10).

If only surface staining was performed, the cells were fixed with 2% paraformaldehyde (Sigma-Aldrich, Taufkirchen, Germany) for 15 min in the dark on ice. For intracellular staining of CD3, cells were permeabilized with 0.5% saponine (Carl Roth, Karlsruhe, Germany) for 10 min at RT. Transcription factor staining and staining of granzyme B, IFN-γ, and IL-17A was performed using the FoxP3/Transcription Factor Staining Buffer Set (Thermo Fisher Scientific, Carlsbad, USA) according to the manufacturer's protocol. After permeabilization, an additional blocking step with dog, rat, and mouse normal serum was done and cells were incubated with antibodies for 30 min at RT. Following acquisition with a BD LSR Fortessa™ flow cytometer (Becton Dickinson, Heidelberg, Germany) cell samples were analyzed using the FlowJo™10 software (Treestar Inc., Ashland, OR, USA). For all flow cytometry plots, biexponential scaling was used which can be retraced in Supplemental Figures 1, 2. Scales were not changed within figures. After exclusion of dead cells, lymphocytes were gated with respect to their size and granularity (Figure 1). Adequate gating was performed by including Fluorescence Minus One (FMO) controls in the experiments. Within FMO controls, the antibody of interest is replaced by its isotype control (all purchased from Thermo Fisher Scientific, Carslbad, USA; or Biolegend, San Diego, USA), whereas all other specific antibodies of the staining panel are included. As suggested by Roederer, for samples with a low number of events only signals with a comparable distribution of fluorescence as appropriate positive controls were assessed as positive (38).

### Statistical Analysis

Statistical analysis of data was done using Graph Pad Prism 5.01 (San Diego, CA, USA) software. To test for normality, Kolmogorov-Smirnov test (with Dallal-Wilkinson-Lillie for *p*-value) was applied. Normally distributed data sets are presented with the mean. For comparison of two normally distributed and independent groups, the unpaired Student's *t*-test (two-tailed) was used, whereas differences between more than two groups were analyzed by One-way analysis of variance (ANOVA) with Bonferroni *post-hoc* test. Nonparametric data are shown with the median. In this case, multiple comparisons were performed by use of the Kruskal-Wallis H test with Dunn's post-test. Comparison of two independent groups was performed using the Mann-Whitney U test (two-tailed). The level of confidence for significance is shown in figure legends.

## Results

### High Frequencies of CD4^−^CD8α^−^ Double-Negative T Cells Can Be Found Within Canine TCRαβ^+^ T Cells of Peripheral Blood, Lymphatic, and Non-lymphatic Organs

CD4^−^CD8α^−^ double-negative (dn) T cells in canine species have been observed in former studies (18, 19, 25), but an in-depth characterization of these cells is still missing. Here we analyze the distribution of CD4^−^CD8α^−^ dn T cells within several lymphatic and non-lymphatic organs of the dog, i.e., peripheral blood mononuclear cells (PBMC), mesenteric (mLN) and tracheobronchial (tLN) lymph nodes, spleen, Peyer's patches (PP), and lung. Viable lymphocytes were gated on either TCRαβ^+^ or TCRγδ^+^ T cells (Figures 1A–E). Regarding the large TCRαβ^+^ lymphocyte subset [mean 79.2% of all lymphocytes (PBMC), Supplemental Figure 3], we were surprised to find a substantial proportion (median values up to 15%) of TCRαβ^+^ cells expressing neither CD4 nor CD8α, with highest frequencies in lung (median 15%), and lowest frequencies in Peyer's patches (median 7.5%, Figures 1D,F,G). Within PBMC, the frequency of TCRαβ^+^CD4^−^CD8α^−^ dn T cells corresponds to about one third of the frequency of TCRαβ^+^CD4^+^ and about half of the frequency of TCRαβ^+^CD8α^+^ single-positive (sp) T cells (i.e., median 14.4% of all TCRαβ^+^ T cells, Supplemental Figures 4A,B). This finding is in clear contrast to other species, like swine, humans, or mice, where TCRαβ^+^CD4^−^CD8α^−^ dn T cells of peripheral blood only comprise a very small proportion (up to 5%) of all T cells (39–41).

As expected, frequencies of TCRγδ^+^ T cells were rather low in all analyzed organs (mean frequencies 0.5–4.6%; Figure 1E, Supplemental Figure 3) which is in line with data by Faldyna et al. for PBMC, spleen, and lymph nodes (16) and characterizing the dog as “γδ T cell low species.” Within this small TCRγδ^+^ cellular population, we did not observe CD4^+^ sp or CD4^+^CD8α^+^ double-positive T cells, but CD8α^+^ sp and about 20–80% CD4^−^CD8α^−^ dn T cells (Figures 1E,H,I, Supplemental Figures 4C,D).

### Canine TCRαβ^+^CD4^−^CD8α^−^ Double-Negative T Cells Show Features of Effector Cells

**Figure 2:**
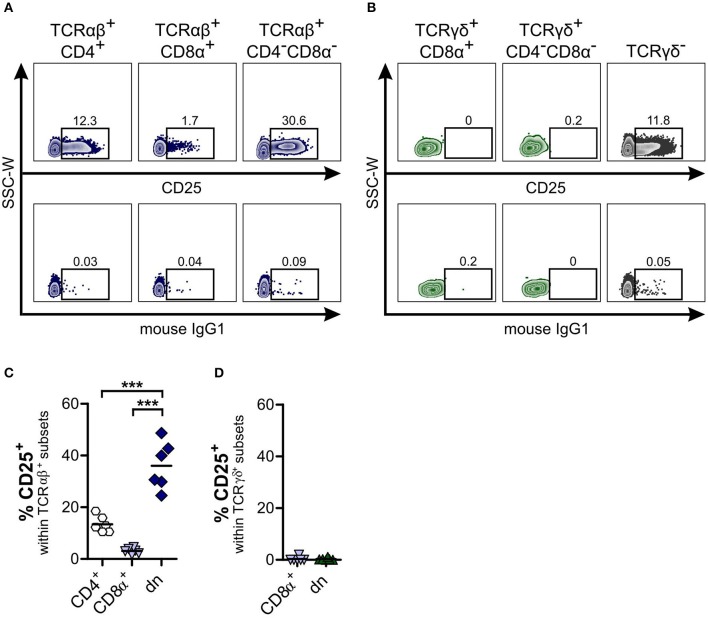
About one third of canine TCRαβ^+^CD4^−^CD8α^−^ double-negative (dn) T cells is activated. Subpopulations of αβ and γδ T cells within PBMC were analyzed for CD25 expression. **(A,B)** CD25 staining of TCRαβ^+^ **(A)** resp. TCRγδ^+^ **(B)** T cell subsets of peripheral blood mononuclear cells (PBMC) in representative zebra plots (upper panels). Gating was performed according to Fluorescence Minus One (FMO) controls (lower panels). For γδ T cells, TCRγδ^−^ cells were analyzed for CD25 expression as staining control (**B**, right panel). **(C,D)** CD25 expression of αβ and γδ T cells was quantified. Shown are frequencies of TCRαβ^+^ (*n* = 6; CD4^+^ sp, white hexagons; CD8α^+^ sp, light blue triangles; CD4^−^CD8α^−^ dn, dark blue diamonds) **(C)**, and TCRγδ^+^ (*n* = 6; CD8α^+^ sp, light blue triangles; CD4^−^CD8α^−^ dn, green triangles) **(D)** subsets expressing CD25. For **(C,D)**, each symbol represents one single dog, the horizontal bars indicate mean values. Statistical analysis was performed by One-way analysis of variance (ANOVA) with Bonferroni's Multiple Comparison Test (***p < 0.001).

To determine the phenotype and differentiation status of canine TCRαβ^+^CD4^−^CD8α^−^ dn T cells, we were interested in surface expression of the activation marker CD25. In contrast to human TCRαβ^+^CD4^−^CD8α^−^ dn T cells (40, 42), a high proportion (mean 36.02%) of canine TCRαβ^+^CD4^−^CD8α^−^ dn T cells of PBMC expresses CD25. Compared to TCRαβ^+^CD4^+^ and TCRαβ^+^CD8α^+^ sp T cells, frequencies of CD25 expression are significantly increased in the TCRαβ^+^CD4^−^CD8α^−^ dn T cell subpopulation (Figures 2A,C). Besides, only TCRαβ^+^CD4^−^CD8α^−^ dn T cells express CD25, whereas TCRγδ^+^CD4^−^CD8α^−^ dn T cells are CD25^−^ (Figures 2B,D).

**Figure 3:**
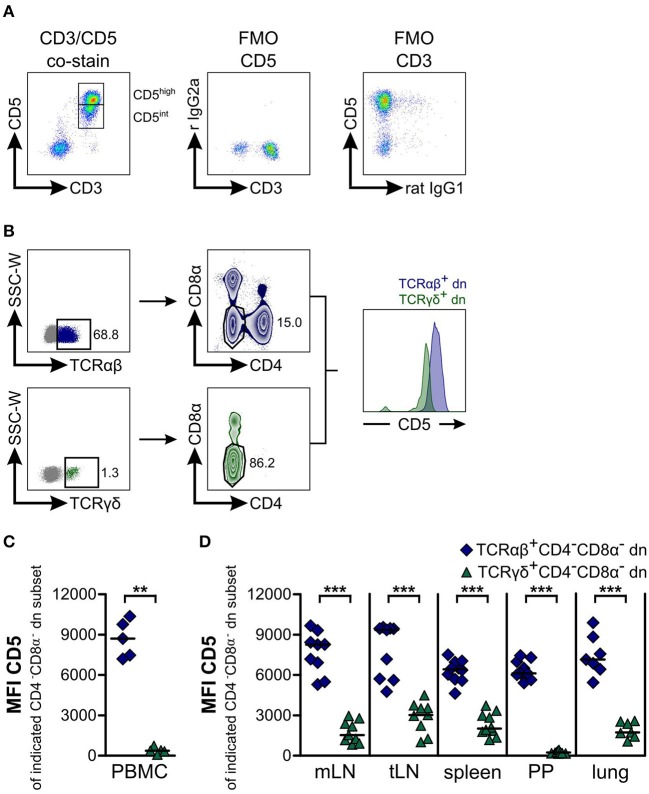
Most TCRαβ^+^CD4^−^CD8α^−^ double-negative (dn) T cells express high levels of CD5, whereas TCRγδ^+^CD4^−^CD8α^−^ dn T cells are CD5^intermediate^. **(A)** Canine peripheral blood mononuclear cells (PBMC) were stained for CD5 and CD3 expression. A representative pseudocolor plot of PBMC gated on living lymphocytes shows co-expression of CD3 and CD5 identifying the population as T cells. CD5^+^ T cells can be divided into CD5^high^ and CD5^intermediate^ (CD5^int^) subpopulations (left panel). Fluorescence Minus One (FMO) controls prove specific staining of antibodies (middle and right panel) (r IgG2a: rat IgG2a). **(B)** Representative plots of canine lung cells showing CD5 expression of TCRαβ^+^CD4^−^CD8α^−^ (dark blue) and TCRγδ^+^CD4^−^CD8α^−^ (green) dn T cells. **(C,D)** Mean Fluorescence Intensity (MFI) of CD5 expression by CD4^−^CD8α^−^ dn T cells pregated on TCRαβ (dark blue diamonds) or TCRγδ (green triangles) is shown. Each symbol represents one single dog, the horizontal bars indicate median values. Statistical analysis was performed by Mann-Whitney U test (two-tailed; **p < 0.01, ***p < 0.001). Peripheral blood (*n* = 5) was taken from another group of Beagle dogs than tissues (*n* = 7–10).

In addition, we wished to analyze expression of CD5 by canine CD4^−^CD8α^−^ dn T cells and to compare TCRαβ^+^ with TCRγδ^+^CD4^−^CD8α^−^ dn T cell subsets. CD5 is primarily expressed on T cells and can be used as T cell marker (43). Moreover, similar to WC1 expressed on bovine γδ T cells, CD5 is composed of scavenger receptor cysteine-rich protein domains characteristic for members of the CD163 family (44, 45). Co-staining of CD3 with CD5 revealed that CD3^+^ T cells of PBMC express CD5 either at a high (CD5^high^) or at an intermediate level (CD5^int^, Figure 3A). Interestingly, the mean fluorescence intensity (MFI) for CD5 of TCRγδ^+^CD4^−^CD8α^−^ dn T cells is significantly decreased in comparison with TCRαβ^+^CD4^−^CD8α^−^ dn T cells (Figures 3C,D) defining most TCRγδ^+^CD4^−^CD8α^−^ dn T cells as a CD5^int^ subset. Of note, a very small proportion of TCRγδ^+^CD4^−^CD8α^−^ dn T cells does not express CD5 (Figure 3B). In conclusion, CD5^high^CD4^−^CD8α^−^ dn cells can be assumed to be TCRαβ^+^. This conclusion was also confirmed by analyzing CD5^high^CD4^−^CD8α^−^ dn vs. TCRαβ^+^CD4^−^CD8α^−^ dn PBMC, as mean frequencies of CD25 expression by both T cell subpopulations were very similar (Supplemental Figures 5A–D).

Regarding CD25 expression of CD5^high^CD4^−^CD8α^−^ dn T cells in tissues, we observed intermediate (lymph nodes) to high proportions (spleen, Peyer's patches, lung) positive for the activation marker CD25. Interestingly, highest frequencies (up to 60% on average) of CD25-positive CD5^high^CD4^−^CD8α^−^ dn T cells could be found within lung tissue (Supplemental Figures 5E–G).

### CD4^−^CD8α^−^ Double-Negative TCRαβ^+^ and TCRγδ^+^ T Cells Differ in Expression of FoxP3, IFN-γ, and IL-17A, but Share High GATA-3 Expression

**Figure 4:**
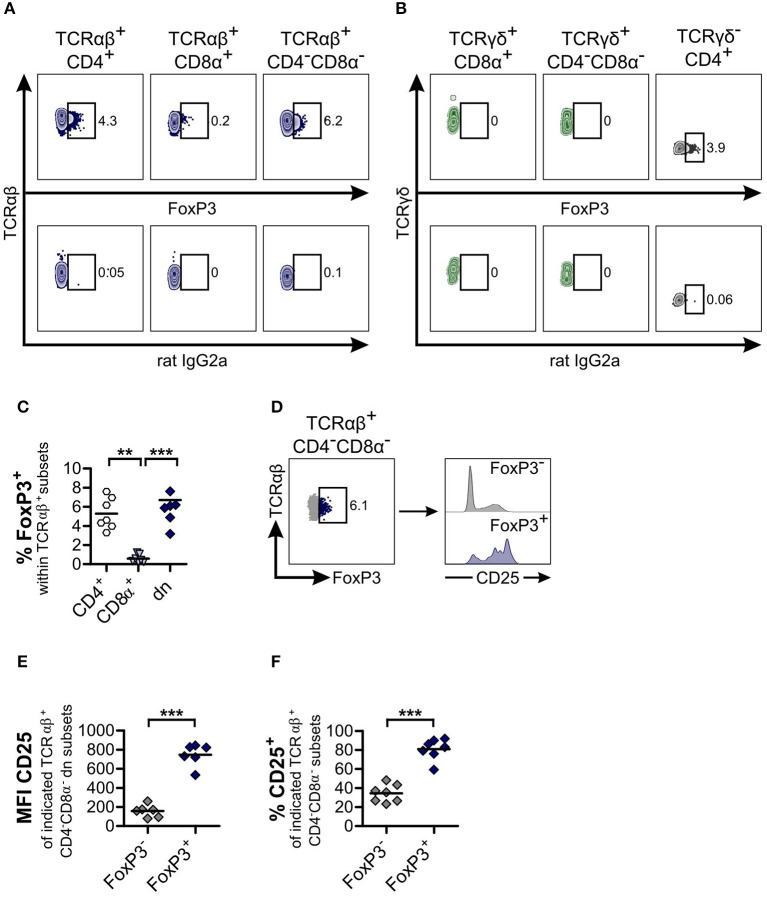
TCRαβ^+^CD4^−^CD8α^−^ double-negative (dn) T cells contain as many FoxP3^+^ cells as TCRαβ^+^CD4^+^ single-positive (sp) T cells. **(A,B)** TCRαβ^+^ **(A)** resp. TCRγδ^+^ **(B)** T cell subsets of peripheral blood mononuclear cells (PBMC) were stained for FoxP3 expression. Shown are representative zebra plots of the corresponding T cell subsets (upper panels). Gating was performed according to Fluorescence Minus One (FMO) controls (lower panels). For γδ T cells, TCRγδ^−^CD4^+^ T cells were analyzed for FoxP3 expression as staining control (**B**, right panel). **(C)** Frequencies of FoxP3^+^ cells within TCRαβ^+^ subsets: CD4^+^ sp (white hexagons), CD8α^+^ sp (light blue triangles), and CD4^−^CD8α^−^ dn (dark blue diamonds) T cells from PBMC. Each symbol represents one individual dog, the horizontal bars indicate mean values (*n* = 7). Statistical analysis was performed by One-way analysis of variance (ANOVA) with Bonferroni‘s Multiple Comparison Test (**p < 0.01, ***p < 0.001). **(D)** Co-staining of CD25 and FoxP3 was performed. CD25 expression of TCRαβ^+^FoxP3^−^ (gray) and TCRαβ^+^FoxP3^+^ (blue) dn T cells is depicted. Shown are representative plots of one dog out of seven. **(E,F)** Mean Fluorescence Intensity (MFI) **(E)** and frequencies **(F)** of CD25 expression of FoxP3^−^ (gray diamonds) and FoxP3^+^ (blue diamonds) dn T cells are shown. For (F), gating on CD25^+^ cells was performed as shown in Figure 2. Each symbol represents one single dog, the horizontal bars indicate mean values (*n* = 7). Statistical analysis was performed by unpaired Student‘s *t*-test (two-tailed, ***p < 0.001).

As TCRαβ^+^CD4^−^CD8α^−^ dn T cells of mice and humans are involved in modulating immune responses (40, 42, 46), and given the high frequencies of CD25 expression of canine TCRαβ^+^CD4^−^CD8α^−^ dn T cells (see Figure 2), we were especially interested in FoxP3 expression of these cells. For TCRαβ^+^CD4^−^CD8α^−^ dn T cells of PBMC, FoxP3 expression could be detected at comparable levels as for their TCRαβ^+^CD4^+^ single-positive counterparts (mean ~6.7%, Figures 4A,C). On the contrary, TCRγδ^+^CD4^−^CD8α^−^ dn T cells of PBMC are FoxP3-negative (Figure 4B).

As expected, the mean fluorescence intensity of CD25 within the FoxP3 expressing TCRαβ^+^CD4^−^CD8α^−^ dn subset was higher than within the FoxP3^−^ counterpart (Figures 4D,E). However, a substantial proportion (mean 34.4%) of the latter subset is CD25^+^, corresponding to an effector phenotype (Figure 4F).

In addition, we observed FoxP3 expression of CD5^high^CD4^−^CD8α^−^ dn T cells in tissues. Median frequencies vary between 1% in mesenteric lymph node and ~3.2% in spleen (Supplemental Figure 6).

**Figure 5:**
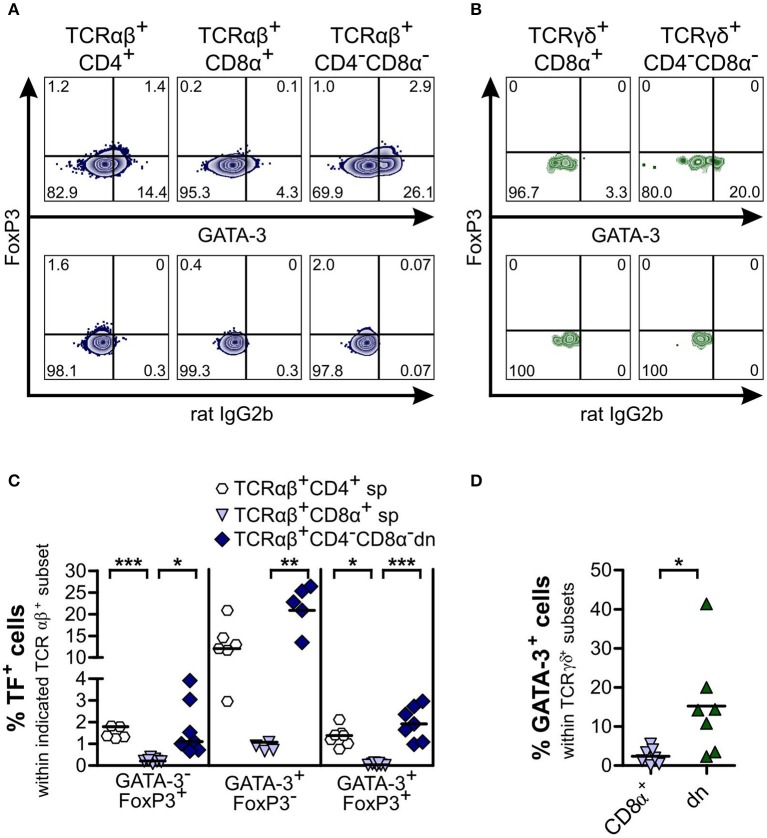
Subsets of CD4^−^CD8α^−^ double-negative (dn) TCRαβ^+^ and TCRγδ^−^ T cells are GATA-3^+^, with a portion of TCRαβ^+^CD4^−^CD8α^−^ dn T cells consisting of GATA-3^+^FoxP3^+^ hybrid cells. Co-staining of FoxP3 and GATA-3 was performed. **(A,B)** Representative zebra plots of αβ **(A)** and γδ T cells **(B)** from PBMC analyzed for expression of FoxP3 and GATA-3 (upper panels). Appropriate gating was confirmed by Fluorescence Minus One controls (lower panels). Numbers in plots represent percentages. **(C)** Quantification of transcription factor (TF) (i.e., GATA-3 and FoxP3) expression of αβ T cell subsets: CD4^+^ sp (white hexagons), CD8α^+^ sp (light blue triangles), and CD4^−^CD8α^−^ dn (dark blue diamonds) T cells (*n* = 7). The horizontal bars indicate median values. Statistical analysis was performed by One-way analysis of variance (ANOVA) with Dunn‘s Multiple Comparison Test (*p < 0.05, **p < 0.01, ***p < 0.001). **(D)** GATA-3^+^ cells within TCRγδ^+^ subsets were quantified: CD8α^+^ sp (light blue triangles) and CD4^−^CD8α^−^ dn (green triangles) T cells (*n* = 7). The horizontal bars indicate mean values. Statistical analysis was performed by unpaired Student‘s *t*-test (two-tailed, *p < 0.05). For **(C,D)**, each symbol represents one individual dog.

Next, we investigated whether CD4^−^CD8α^−^ dn T cells exhibit properties of T helper (Th) 2, Th1, Th17, or cytotoxic T cells by analyzing expression of the transcription factors GATA-3 and T-bet, of the cytokines IFN-γ and IL-17A as well as of the cytotoxicity marker granzyme B. For both αβ and γδ T cells, we observed only low proportions of GATA-3^+^ cells within the CD8α^+^ sp subset. On the other hand, TCRαβ^+^CD4^+^ sp and both (TCRαβ^+^ and TCRγδ^+^) CD4^−^CD8α^−^ dn T cell subsets express GATA-3. Interestingly, frequencies of GATA-3 expressing TCRαβ^+^CD4^−^CD8α^−^ dn T cells were even higher (though not significantly elevated) as compared with conventional TCRαβ^+^CD4^+^ sp T cells (median 20.6% vs. 11.9%). For αβ T cells, a second GATA-3^+^ subset could be identified in CD4^+^ sp and CD4^−^CD8α^−^ dn T cells co-expressing the Treg transcription factor FoxP3 (Figure 5).

**Figure 6:**
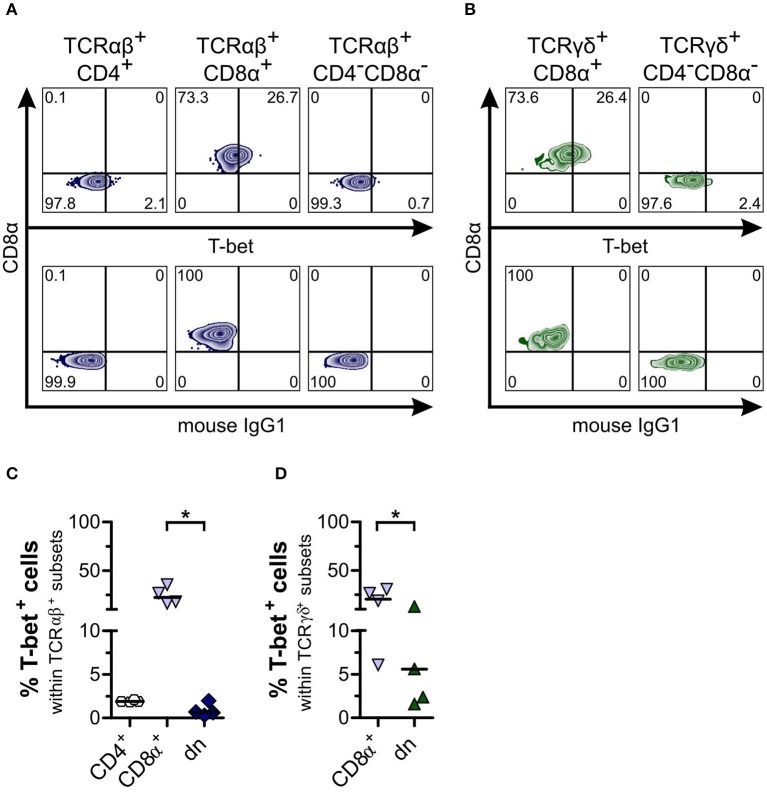
Canine CD4^−^CD8α^−^ double-negative (dn) T cells express low frequencies of T-bet in comparison to CD8α^+^ single-positive (sp) T cells. **(A)** Representative zebra plots illustrating expression of T-bet by TCRαβ^+^ T cells gated on CD4^+^ sp, CD8α^+^ sp, and CD4^−^CD8α^−^ dn PBMC (upper panels). **(B)** TCRγδ^+^ cells including CD8α^+^ sp and CD4^−^CD8α^−^ dn subsets were analyzed for T-bet expression shown by representative zebra plots (upper panels). For **(A,B)**, appropriate gating was confirmed by internal and Fluorescence Minus One controls (lower panels). **(C)** Frequencies of T-bet expression by TCRαβ^+^CD4^+^ sp (white hexagons), TCRαβ^+^CD8α^+^ sp (light blue triangles), and TCRαβ^+^CD4^−^CD8α^−^ dn (dark blue diamonds) T cells of PBMC are depicted. The horizontal bars indicate median values (*n* = 4). Statistical analysis was performed by One-way analysis of variance (ANOVA) with Dunn's Multiple Comparison Test (*p < 0.05). **(D)** Quantification of T-bet expression within TCRγδ^+^ T cell subsets (CD8α^+^, light blue triangles; CD4^−^CD8α^−^ dn, green triangles) is shown. The horizontal bars indicate mean values (*n* = 4). Statistical analysis was performed by unpaired Student‘s *t*-test (two-tailed, *p < 0.05).

As shown previously for dogs, the transcription factor T-bet is constitutively expressed by canine CD8α^+^ sp, but only at a very low degree by CD4^+^ sp peripheral T cells (27). Similar to the latter, we observed significantly lower T-bet expression of both TCRαβ^+^ and TCRγδ^+^CD4^−^CD8α^−^ dn T cells of canine PBMC in comparison to CD8α^+^ sp T cells (Figure 6). For CD5^high^CD4^−^CD8α^−^ dn T cells of tissues, low T-bet expression in comparison to CD8α^+^ sp T cells was found as well (Supplemental Figure 7).

**Figure 7:**
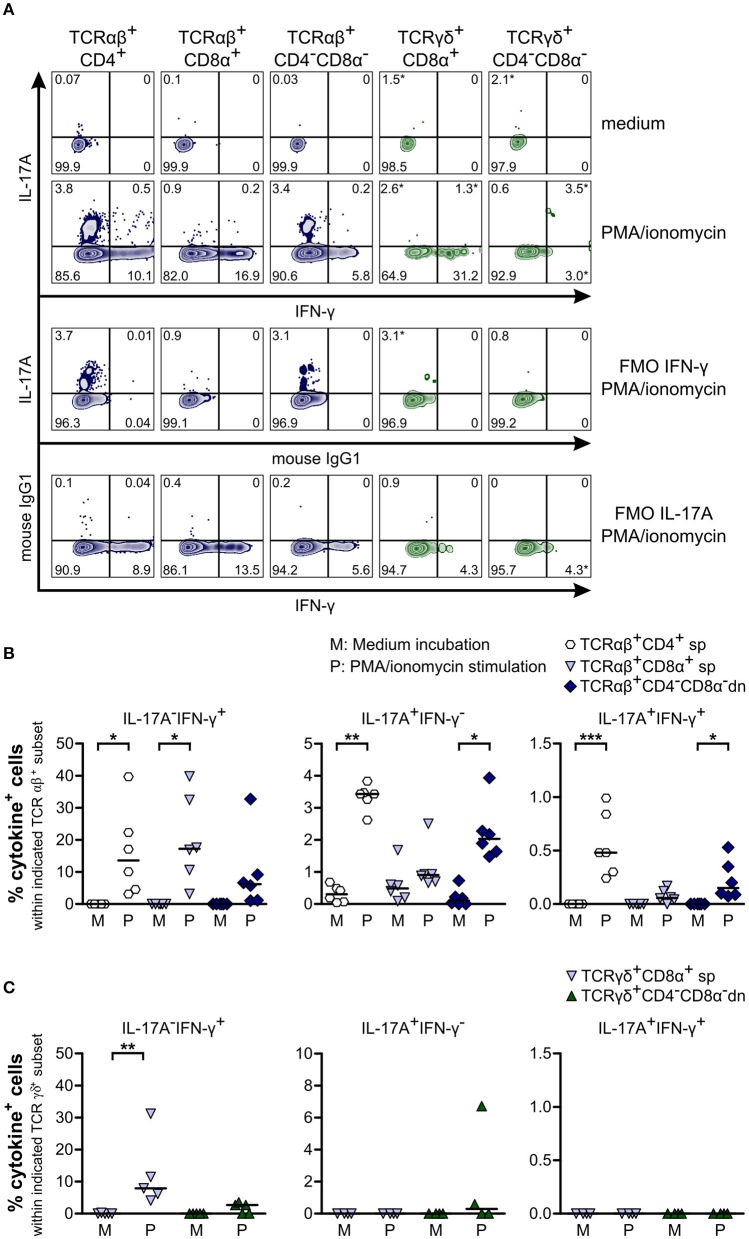
Canine TCRαβ^+^CD4^−^CD8α^−^ but not TCRγδ^+^CD4^−^CD8α^−^ double-negative (dn) T cells produce IFN-γ and IL-17A upon PMA/ionomycin stimulation. **(A)** Representative zebra plots illustrating IL-17A and IFN-γ production by canine TCRαβ^+^ and TCRγδ^+^ T cell subsets after 4 h of PMA/ionomycin stimulation. Adequate gating was performed according to medium (upper panels) and Fluorescence Minus One (FMO) controls. Frequencies marked with * were disregarded based on the low MFI of only single data points according to Roederer (38). **(B)** Quantification of IL-17A and IFN-γ production by αβ T cell subsets after 4 h of medium (M) incubation or stimulation with PMA/ionomycin (P): CD4^+^ sp (white hexagons), CD8α^+^ sp (light blue triangles), and CD4^−^CD8α^−^ dn (dark blue diamonds) T cells. The horizontal bars indicate median values. Statistical analysis was performed by One-way analysis of variance (ANOVA) with Dunn‘s Multiple Comparison Test (*p < 0.05, **p < 0.01, ***p < 0.001). Three independent experiments with *n* = 6 dogs in total were performed. **(C)** Quantification of IL-17A and IFN-γ production by TCRγδ^+^CD8α^+^ sp (light blue triangles) and TCRγδ^+^CD4^−^CD8α^−^ dn (green triangles) T cells as described in **(B)**. The horizontal bars indicate median values. Statistical analysis was performed by One-way analysis of variance (ANOVA) with Dunn‘s Multiple Comparison Test (**p < 0.01). Three independent experiments with *n* = 4–5 dogs in total were performed.

To study cytokine production by CD4^−^CD8α^−^ dn T cells, we stimulated PBMC with PMA/ionomycin and looked for production of IFN-γ and IL-17A. IFN-γ was elevated in TCRαβ^+^CD4^−^CD8α^−^ dn T cells as compared with the medium control sample. Moreover, significantly increased IFN-γ production was found for their CD4^+^ and CD8α^+^ sp counterparts (Figures 7A,B). For γδ T cells, IFN-γ could only be observed within TCRγδ^+^CD8α^+^ sp T cells, whereas this cytokine was barely detectable within TCRγδ^+^CD4^−^CD8α^−^ dn T cells (Figures 7A,C). Interestingly, TCRαβ^+^CD4^−^CD8α^−^ dn T cell subsets produce the pro-inflammatory cytokine IL-17A upon PMA/ionomycin stimulation, similar to TCRαβ^+^CD4^+^ sp, and in contrast to TCRαβ^+^CD8α^+^ sp T cells (Figures 7A,B). Contrary to murine, human, porcine and bovine γδ T cells (47–51), canine γδ T cells (CD8α^+^ sp and CD4^−^CD8α^−^ dn) do not appear to express IFN-γ or IL-17A upon stimulation with PMA/ionomycin (Figures 7A,C).

**Figure 8:**
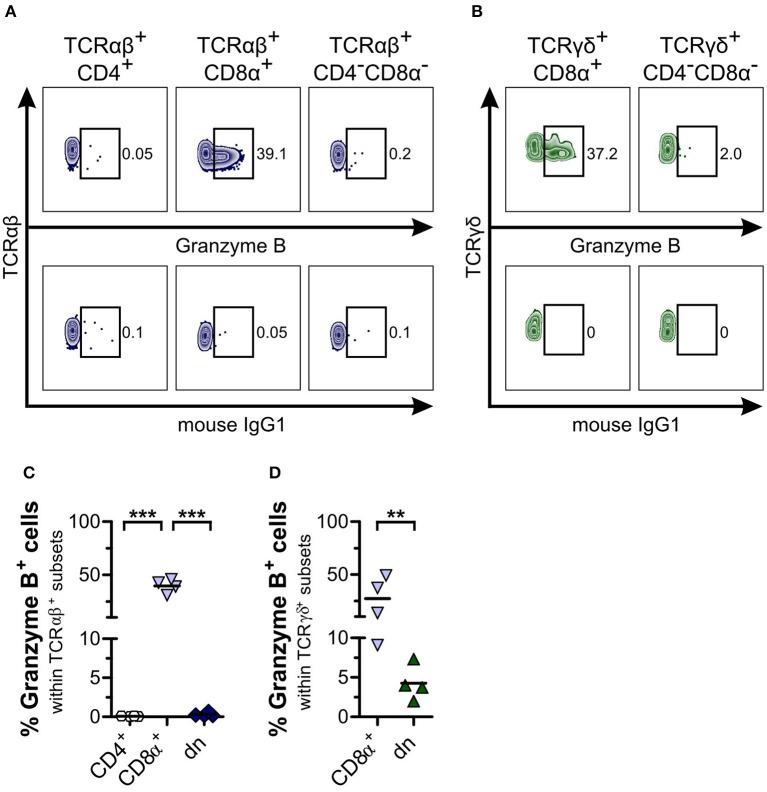
CD4^−^CD8α^−^ double-negative (dn) T cells are mainly granzyme B^−^. **(A,B)** Representative zebra plots illustrating expression of granzyme B by TCRαβ^+^ **(A)** resp. TCRγδ^+^ **(B)** T cell subsets of PBMC (upper panels). Appropriate gating was confirmed by internal and Fluorescence Minus One controls (lower panels). Numbers in plots imply percentages. **(C,D)** The mean frequencies of granzyme B expression by TCRαβ^+^ (CD4: white hexagons, CD8α^+^: light blue triangles, dn: dark blue diamonds) **(C)** resp. TCRγδ^+^ (CD8α^+^: light blue triangles, dn: green triangles**) (D)** T cell subpopulations of PBMC are depicted. Each symbol represents one single dog (*n* = 4). For **(C)**, statistical analysis was performed by One-way analysis of variance (ANOVA) with Bonferroni‘s Multiple Comparison Test (***p < 0.001). For **(D)**, the unpaired Student‘s *t*-test (two-tailed, **p < 0.01) was applied.

Finally, we looked for expression of the cytotoxic molecule granzyme B within TCRαβ^+^ and TCRγδ^+^CD4^−^CD8α^−^ dn T cell subsets. For TCRαβ^+^CD4^−^CD8α^−^ dn PBMC, no granzyme B expression could be detected in the resting state (Figures 8A,C). Regarding γδ T cells, we observed granzyme B expression by both CD8α^+^ sp and CD4^−^CD8α^−^ dn T cells, even though granzyme B expression of CD4^−^CD8α^−^ dn T cells was significantly decreased in comparison with CD8α^+^ sp γδ T cells (Figures 8B,D). For tissues, too, only low frequencies of granzyme B were observed within the CD5^high^CD4^−^CD8α^−^ dn fraction (Supplemental Figure 8).

Taken together, these data show that canine TCRαβ^+^CD4^−^CD8α^−^ dn T cells are a large population within all TCRαβ^+^ cells. They comprise surprisingly high numbers of effector T cells and subsets expressing FoxP3 and/or GATA-3, along with IFN-γ or IL-17A producing cells. On the other hand, the small subset of γδ T cells consists of CD8α^+^ sp and CD4^−^CD8α^−^ dn T cells with the latter expressing GATA-3 as well as some T-bet and granzyme B but lacking IFN-γ and IL-17A production.

## Discussion

To date, canine extrathymic non-conventional CD4^−^CD8α^−^ double-negative (dn) T cells (CD3^+^, TCRαβ^+^, or TCRγδ^+^) have only been observed in few previous studies where their potential importance was discussed (17–19, 25). To provide the basis for an in-depth understanding of these cells, we undertook a multiparameter flow cytometry analysis of canine TCRαβ^+^ and TCRγδ^+^CD4^−^CD8α^−^ dn T cells revealing their (i) frequency and tissue distribution, (ii) activation state, and (iii) functional potency by analysis of transcription factor (FoxP3, GATA-3, T-bet), cytokine (IFN-γ, IL-17A), and cytotoxicity marker (i.e., granzyme B) expression. The data obtained demonstrate a large subset of peripheral blood and tissue TCRαβ^+^CD4^−^CD8α^−^ dn T cells with surprisingly high activation and an effector phenotype expressing FoxP3 and/or GATA-3. In contrast, canine TCRαβ^+^CD4^−^CD8α^−^ dn T cells hardly express T-bet or granzyme B. With these features they clearly differ from their less numerous TCRγδ^+^CD4^−^CD8α^−^ dn counterparts, and even more from conventional TCRαβ^+^CD8α^+^ sp T cells. On the other hand, we found phenotypic similarities to TCRαβ^+^CD4^+^ sp T cells, even though their activation state is remarkably different from the latter and rather comparable to canine TCRαβ^+^CD4^+^CD8α^+^ double-positive T cells (26, 27, 52).

A majority of canine CD4^−^CD8α^−^ dn T cells was found to express mainly TCRαβ, similar to murine and human CD4^−^CD8α^−^ dn T cells of lymphoid tissues. However, in the dog the proportion in peripheral blood or lymphoid tissues is clearly higher than in mouse or man (40, 41, 53). This of course raises the question related to the function of such a large non-conventional TCRαβ^+^ T cell subset in dogs, but also related to the function of the smaller TCRγδ^+^ cell subset with its remarkably high expression of GATA-3. Answers to these important questions will only be possible after additional CD4^−^CD8α^−^ dn T cell studies have been done in dogs during immune homeostasis or immune activation, e.g., in context with immunization/infection, cancer, autoimmunity, or allergy.

It was surprising to find activation of a high portion of canine TCRαβ^+^CD4^−^CD8α^−^ dn T cells. Similar studies in human or murine TCRαβ^+^CD4^−^CD8α^−^ dn T cells show about 7–10-fold lower portions of CD25 expressing cells in peripheral blood or spleen (40, 54). Of note, within the murine urogenital tract elevated frequencies of activated TCRαβ^+^CD4^−^CD8α^−^ dn T cells have been described (41, 55). Currently the mechanisms driving high activation of canine TCRαβ^+^CD4^−^CD8α^−^ dn T cells in peripheral blood or lymphoid tissues remain elusive. TCRαβ-mediated triggering (leading to loss of naïve status and acquisition of an effector phenotype) and/or pattern recognition receptor (PRR)-dependent stimulation (e.g., at epithelial barriers) may contribute to activation of canine TCRαβ^+^CD4^−^CD8α^−^ dn T cells. The nature of the antigens responsible for TCRαβ-mediated activation and/or the type of PRR as well as the pathogen-associated molecular pattern(s) (PAMPs) for co-stimulation are currently unknown.

Expression of transcription factors has been demonstrated to determine subset-specific T cell function. To this end we analyzed expression of FoxP3, GATA-3, and T-bet by canine CD4^−^CD8α^−^ dn T cells. FoxP3 is the master regulator of conventional regulatory T cells (Treg) (56). Indeed, FoxP3 expression by TCRαβ^+^CD4^−^CD8α^−^ dn T cells reached the same levels as found for classical Treg (i.e., TCRαβ^+^CD4^+^ sp T cells). Evidence for this regulatory potential might be the very recently described increase of peripheral blood CD4^−^CD8α^−^ dn T cells after food allergen-specific sublingual immunotherapy of dogs with adverse food reactions. A potential regulatory function of these cells was discussed, albeit FoxP3 expression was not analyzed (19). GATA-3 expression by Tregs has been shown to play an essential role for Treg function during inflammation but not at steady state (e.g., for recruitment of Tregs to inflamed sites and for maintenance of high levels of FoxP3 expression) (57). Thus, we analyzed potential co-expression of FoxP3 and GATA-3 by TCRαβ^+^CD4^−^CD8α^−^ dn T cells and were able to detect cells with simultaneous expression of FoxP3 and GATA-3. Co-expression of FoxP3 and GATA-3 by a subset of canine TCRαβ^+^CD4^−^CD8α^−^ dn T cells may stabilize their suppressive capacity and prevent conversion into a pro-inflammatory T helper (Th) 17 cell phenotype as has been shown for murine GATA-3-deficient Tregs (58). On the other hand, canine TCRαβ^+^ and TCRγδ^+^CD4^−^CD8α^−^ dn T cells showed a significantly elevated percentage of GATA-3^+^FoxP3^−^ cells as compared with TCRαβ^+^ resp. TCRγδ^+^CD8α^+^ sp T cells. TCRαβ^+^CD4^−^CD8α^−^ dn T cells reached even higher percentages of GATA-3 expression than TCRαβ^+^CD4^+^ sp Th cells. While low GATA-3 expression (e.g., by CD8α^+^ sp T cells) may functionally be attributed to a role of GATA-3-dependent development of T cells (59), the high GATA-3 levels by TCRαβ^+^CD4^+^ sp T cells and in particular by CD4^−^CD8α^−^ dn T cells may reflect the function of GATA-3 as Th2 master regulator (60, 61). Therefore, it is conceivable that canine GATA-3^+^CD4^−^CD8α^−^ dn T cells play a role in type 2 immunity such as anti-parasite responses or in the pathophysiology of allergy. To fully assess a potential type 2 regulatory function of canine GATA-3^+^CD4^−^CD8α^−^ dn T cells, characteristic features of Th2 cells such as production of IL-4, IL-5, or IL-13 need to be studied in future experiments. Besides, to verify a potential suppressive function of canine FoxP3^+^ (GATA-3^+^) TCRαβ^+^CD4^−^CD8α^−^ dn T cells, their potential production of IL-10 and TGF-β as well as their suppressive capacity has to be assessed *in vitro*.

Unfortunately, due to the lack of cross-reactive monoclonal antibodies against canine RORγt, analysis of a potential Th17 transcriptional signature of canine CD4^−^CD8α^−^ dn T cells is currently not possible. However, we found evidence for a potential Th17 phenotype of a TCRαβ^+^CD4^−^CD8α^−^ dn T cell subset by analyzing expression of the cytokine IL-17A. In healthy humans, too, IL-17 has been found to be expressed by TCRαβ^+^CD4^−^CD8α^−^ dn T cells. Increased frequencies of these cells were found in samples of human patients with autoimmune diseases (62–64). Furthermore, canine γδ T cells do not appear to be potent IL-17A producers. This is in clear contrast to murine, human, porcine and bovine γδ T cells (47–49, 51). It should be taken in account that canine γδ T cells might require different stimuli than PMA/ionomycin to produce IL-17, even though PMA/ionomycin was successfully used to stimulate γδ T cells of other species, e.g., swine (49, 65).

Further functional analysis of both subsets (TCRαβ^+^ and TCRγδ^+^) of canine CD4^−^CD8α^−^ dn T cells will help to generate new hypotheses related to their role *in vivo*. Besides, it should be validated whether TCRαβ^+^CD4^−^CD8α^−^ dn T cells acquire cytotoxic potential upon activation despite their lack of granzyme B expression in the resting state.

With the features of CD4^−^CD8α^−^ dn T cell subsets described in our study, it is conceivable that they may have a pivotal function during homeostasis by suppressing exuberant immune responses and during inflammatory diseases of dogs.

## Data Availability Statement

The datasets generated for this study are available on request to the corresponding author.

## Ethics Statement

The animal study was reviewed and approved by the Animal Care and Usage Committee of the Saxony State Office (Landesdirektion Sachsen) in Leipzig, Germany (approval numbers: A 10/14 and A 28/18) (blood samples). Tissue samples: the study was approved by the Regierungspräsidium Darmstadt, Germany, approval number V54-19c 20/15-DA4/Anz.1004.

## Author Contributions

FR and ME: designed experiments, performed experiments, and wrote the manuscript. PM: provided reagents. KR, HB, DB, MB, and GA: wrote the manuscript.

### Conflict of Interest

The authors declare that the research was conducted in the absence of any commercial or financial relationships that could be construed as a potential conflict of interest.
