# Supplementary figures and images for: Distinct Features of Canine Non-conventional CD4−CD8α− Double-Negative TCRαβ+ vs. TCRγδ+ T Cells
Source: Front Immunol. 2019 Nov 22;10:2748. doi: 10.3389/fimmu.2019.02748 (PMC6883510; doi:10.3389/fimmu.2019.02748)

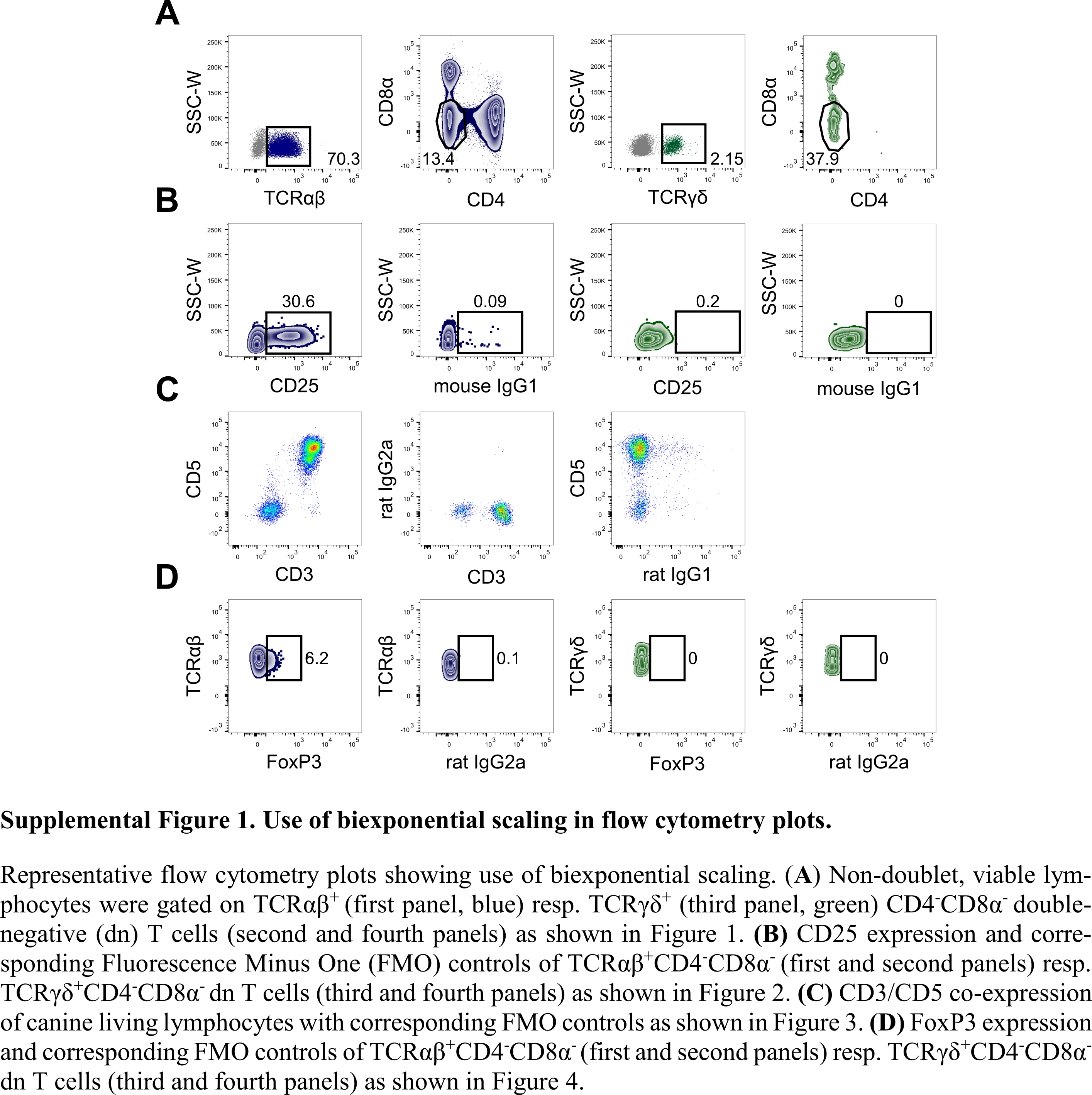

Supplement: Supplementary file 1 [file Image_1.JPEG]

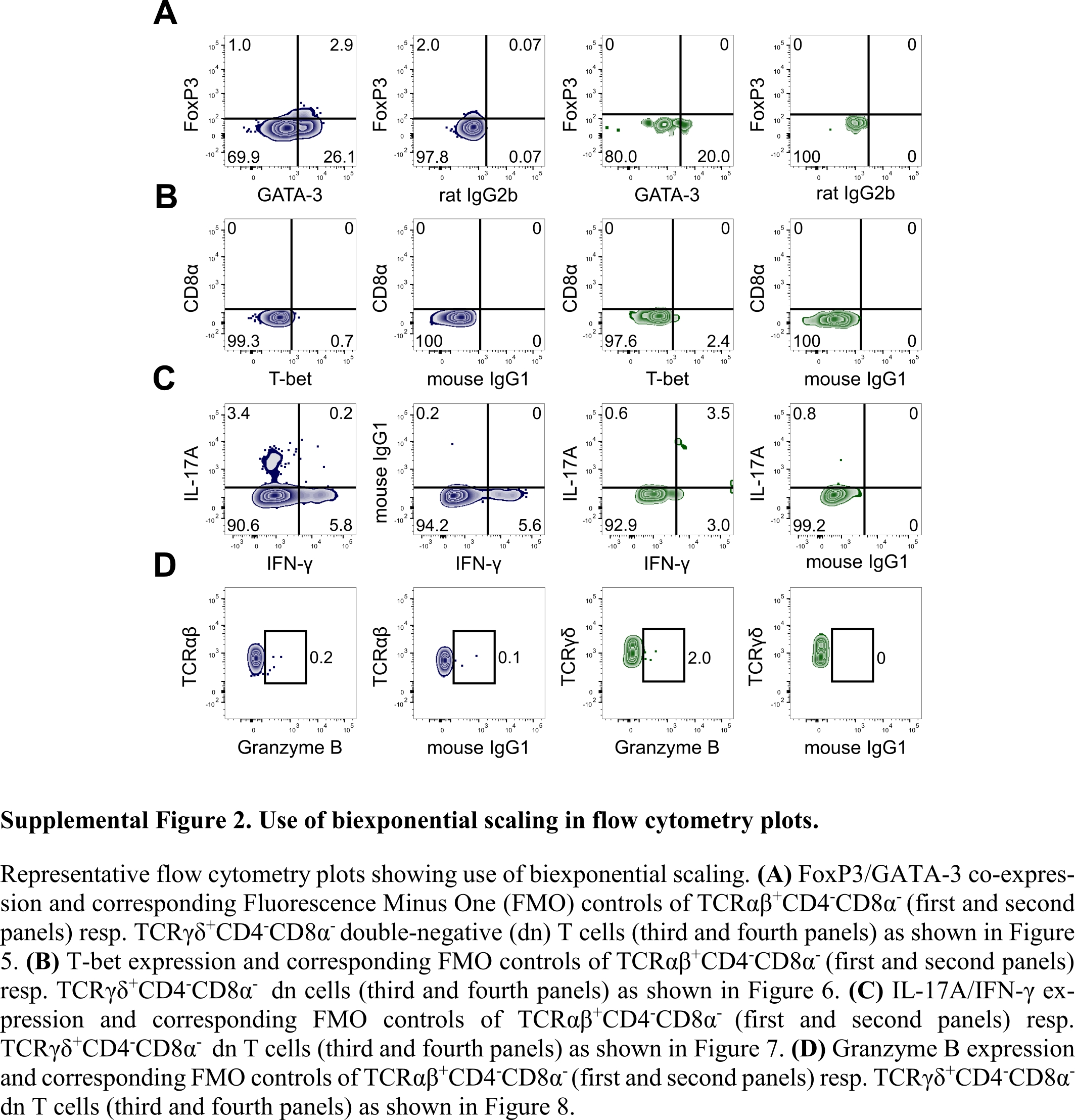

Supplement: Supplementary file 2 [file Image_2.JPEG]

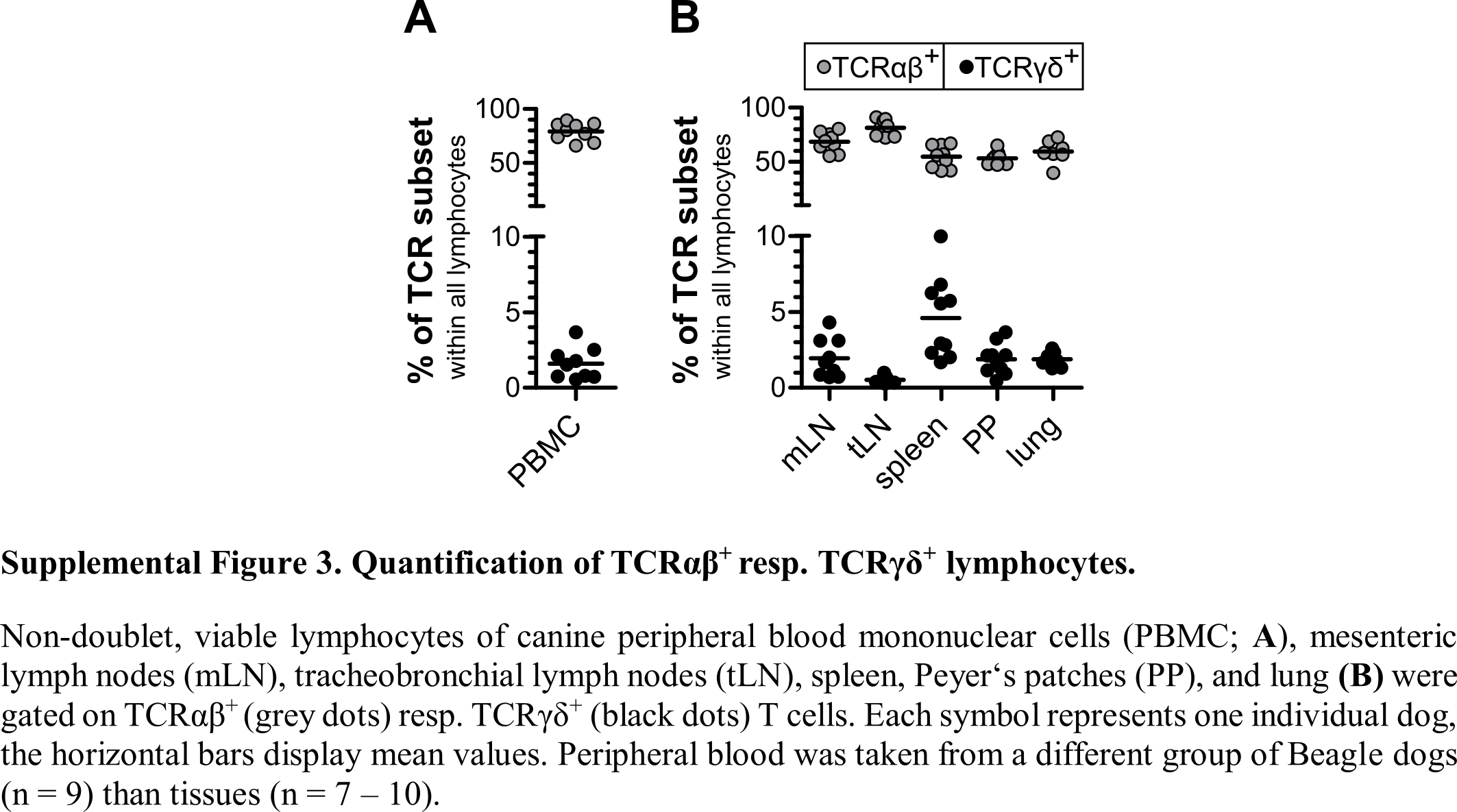

Supplement: Supplementary file 3 [file Image_3.JPEG]

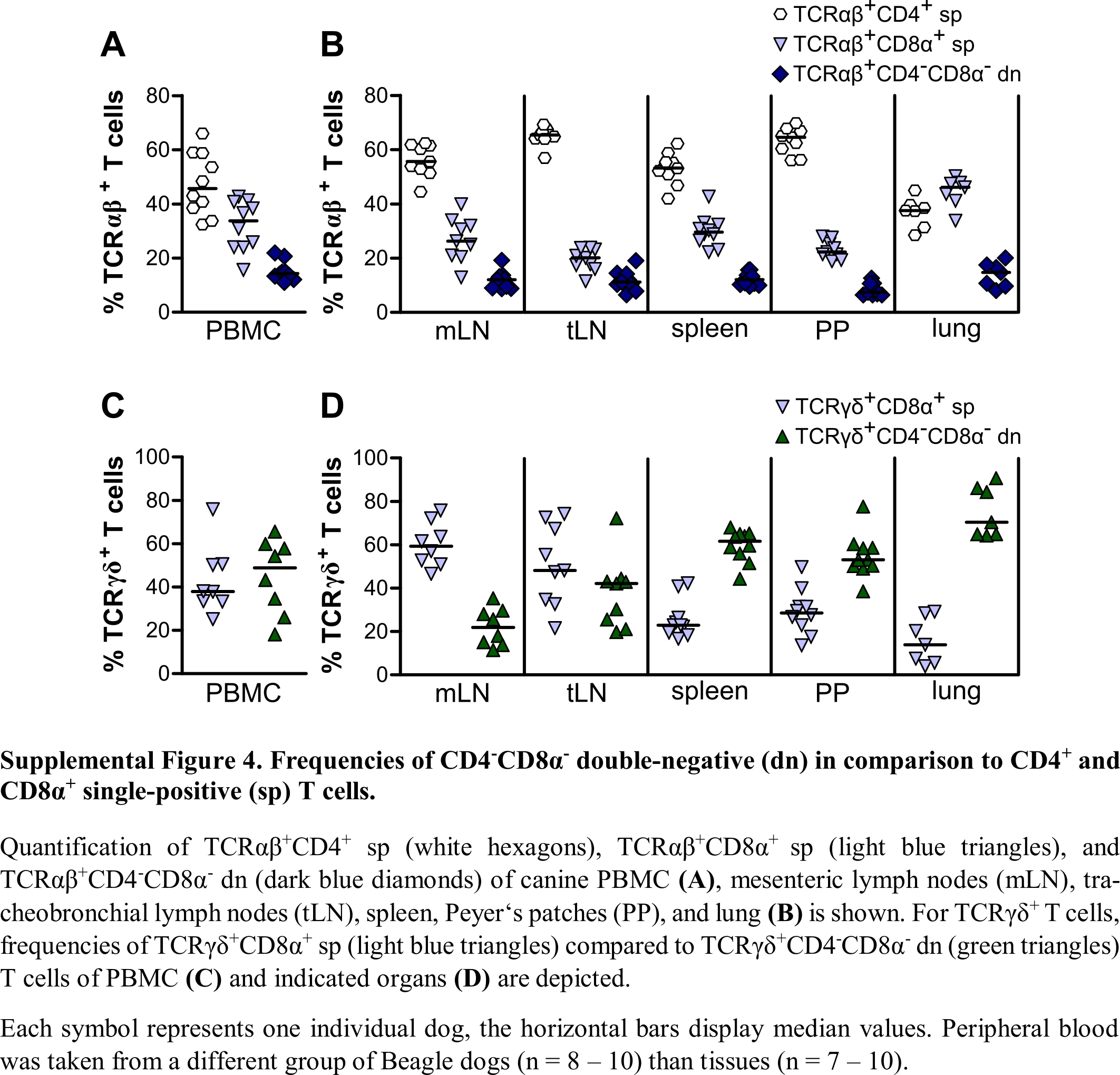

Supplement: Supplementary file 4 [file Image_4.JPEG]

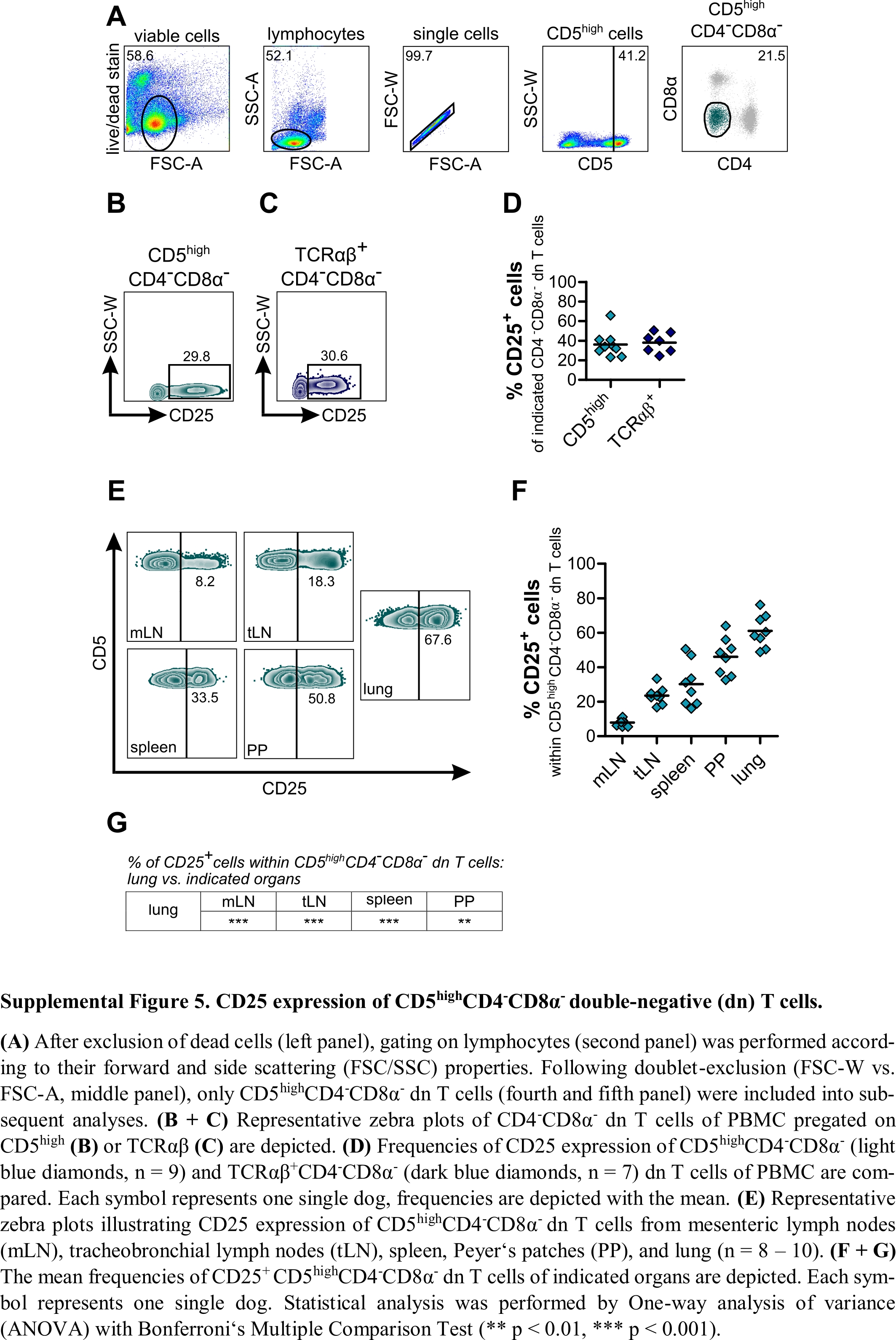

Supplement: Supplementary file 5 [file Image_5.JPEG]

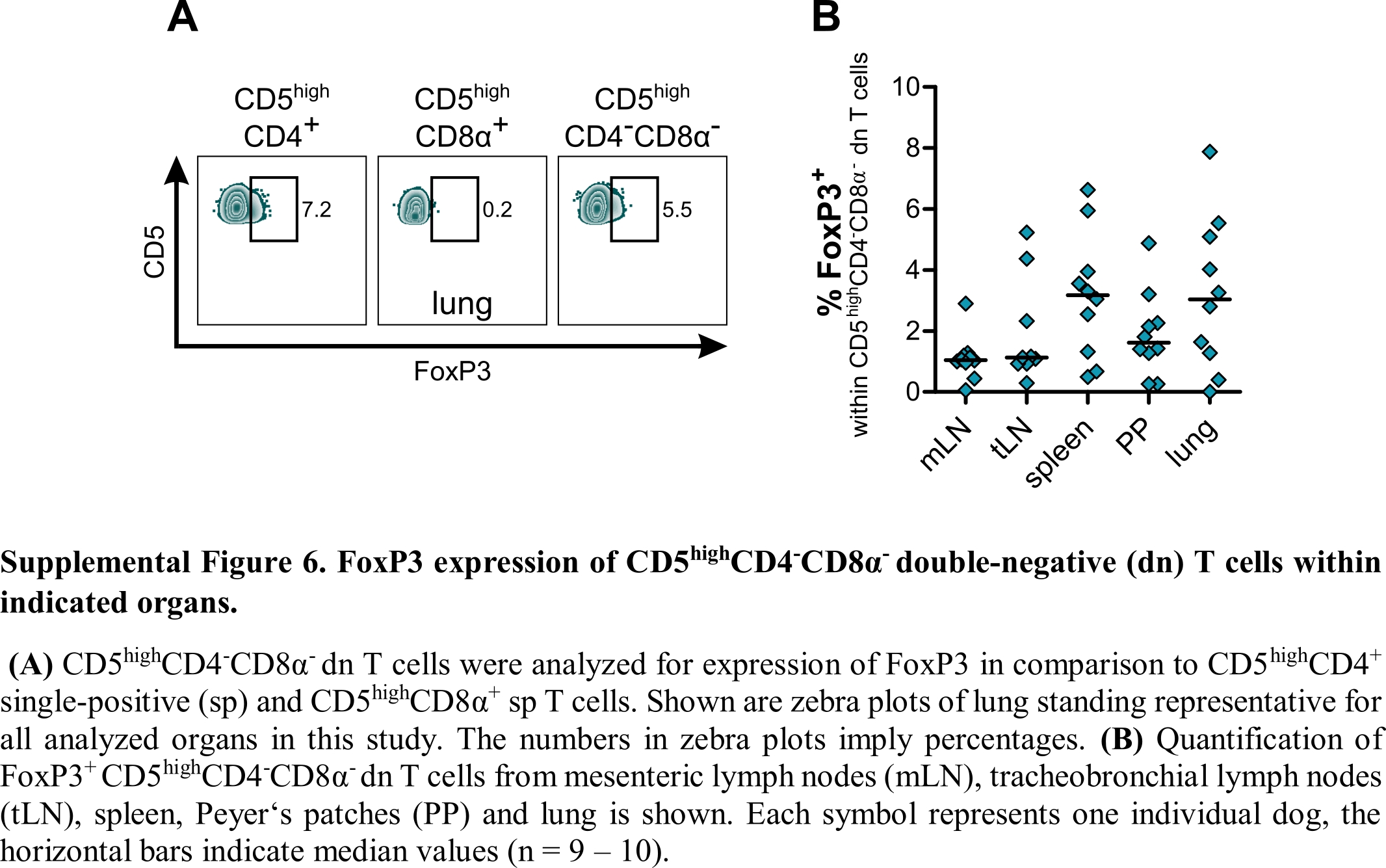

Supplement: Supplementary file 6 [file Image_6.JPEG]

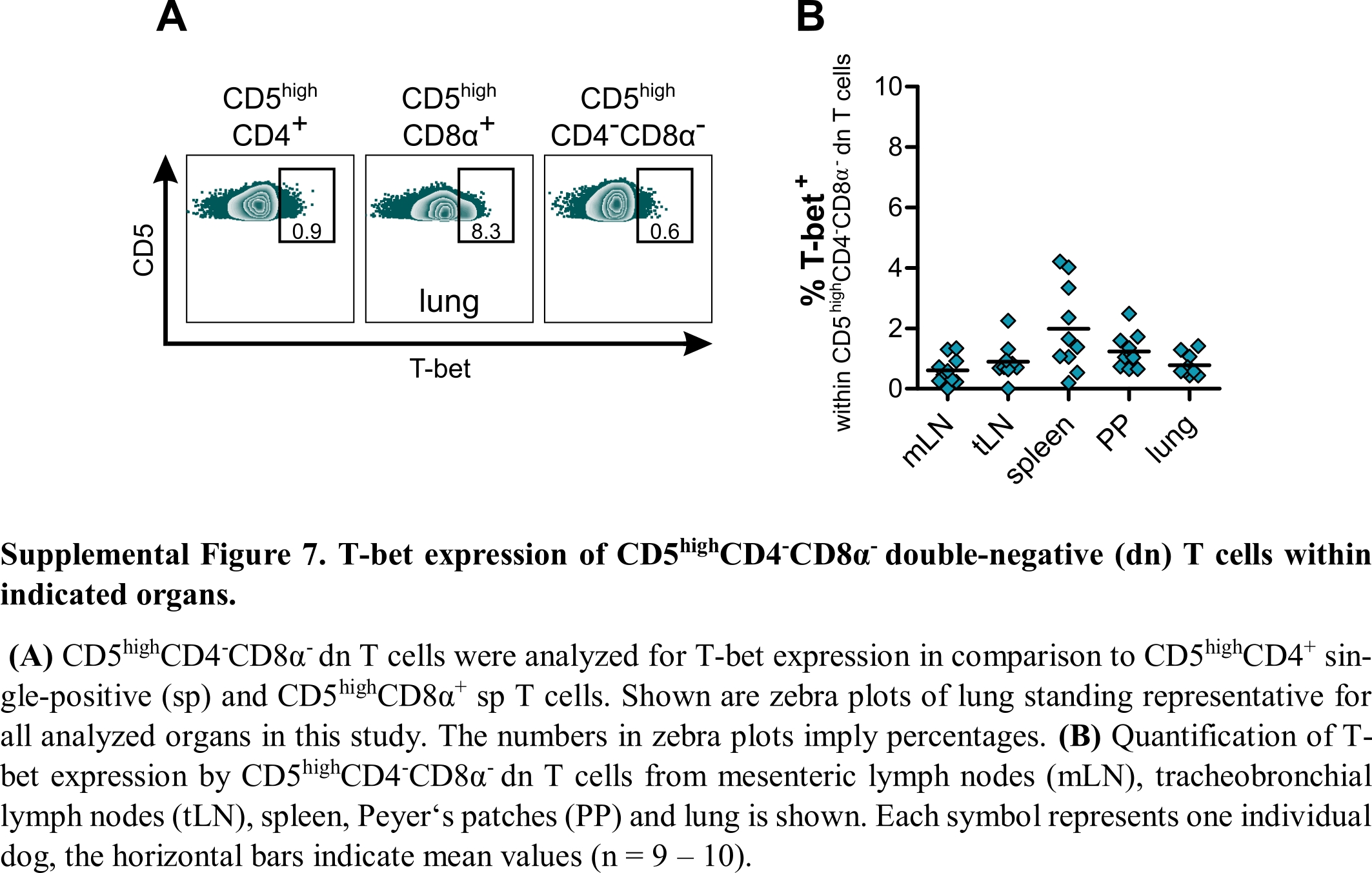

Supplement: Supplementary file 7 [file Image_7.JPEG]

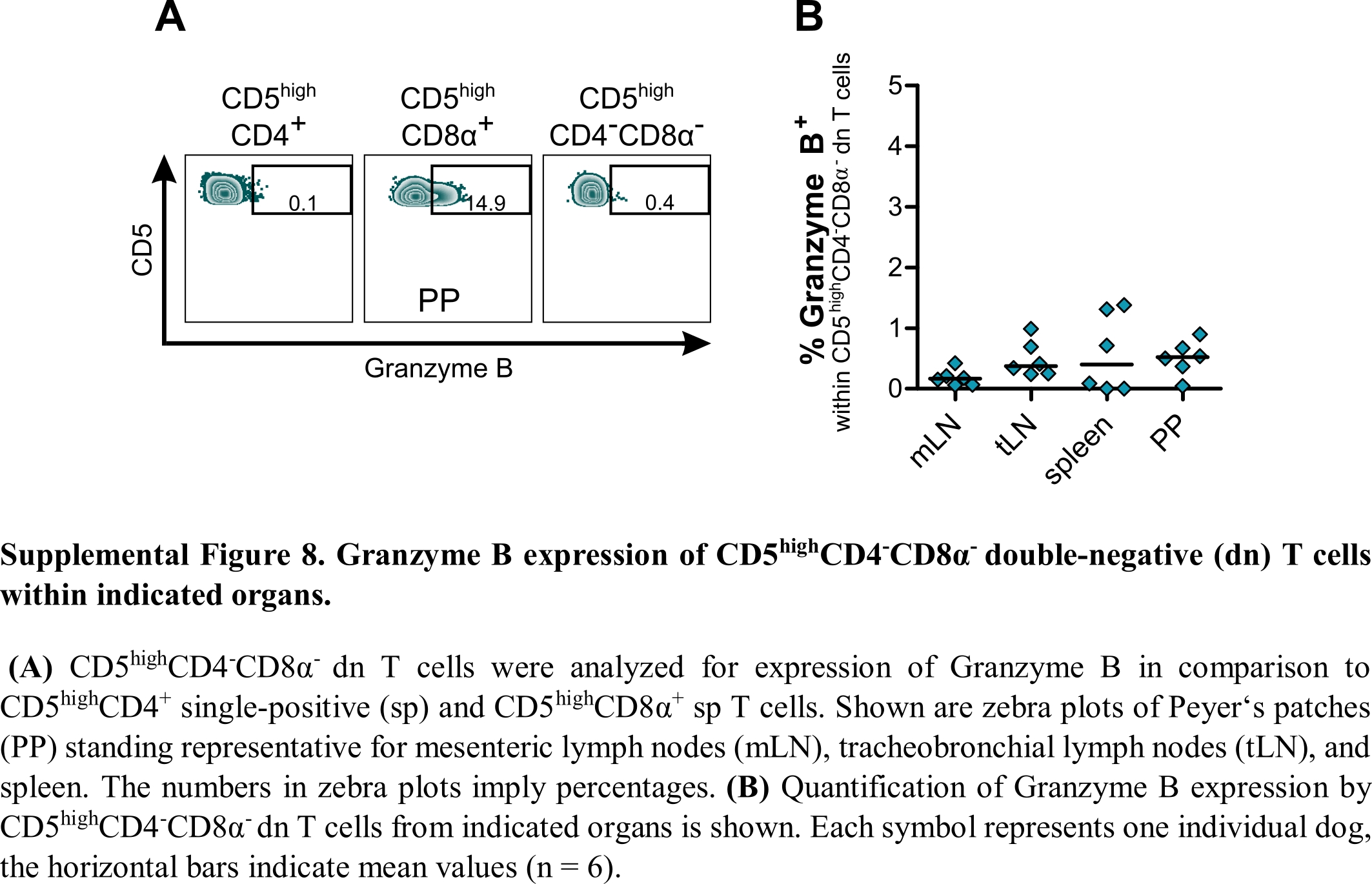

Supplement: Supplementary file 8 [file Image_8.JPEG]
